# A randomized double-blind placebo-controlled clinical trial of nitazoxanide for treatment of mild or moderate COVID-19

**DOI:** 10.1016/j.eclinm.2022.101310

**Published:** 2022-02-28

**Authors:** Jean-François Rossignol, Matthew C. Bardin, Jessica Fulgencio, Dena Mogelnicki, Christian Bréchot

**Affiliations:** a Romark Institute of Medical Research, Tampa, FL, United States; b University of South Florida, College of Medicine, Tampa, FL, United States; c Global Virus Network, United States

## Abstract

**Background:**

There is an urgent need for treatments of mild or moderate COVID-19 in an outpatient setting.

**Methods:**

A randomized double-blind placebo-controlled clinical trial in 36 centers in the U.S. between August 2020 and February 2021 investigated the safety and effectiveness of oral nitazoxanide 600 mg twice daily for five days in outpatients with symptoms of mild or moderate COVID-19 enrolled within 72 h of symptom onset (ClinicalTrials.gov NCT04486313). Efficacy endpoints were time to sustained clinical recovery (TSR, a novel primary endpoint) and proportion of participants progressing to severe illness within 28 days (key secondary).

**Findings:**

1092 participants were enrolled. 379 with laboratory-confirmed SARS-CoV-2 infection were analyzed. In the primary analysis, median (IQR) TSR were 13·3 (6·3, >21) and 12·4 (7·2, >21) days for the nitazoxanide and placebo groups, respectively (*p* = 0·88). 1 of 184 (0·5%) treated with nitazoxanide progressed to severe illness compared to 7 of 195 (3·6%) treated with placebo (key secondary analysis, odds ratio 5·6 [95% CI 0·7 - 46·1], relative risk reduction 85%, *p* = 0·07). In the pre-defined stratum with mild illness at baseline, nitazoxanide-treated participants experienced reductions in median TSR (3·1 days, *p* = 0·09) and usual health (5·2 days, *p* < 0·01) compared to placebo. Nitazoxanide was safe and well tolerated.

**Interpretation:**

Further trials with larger numbers are warranted to evaluate efficacy of nitazoxanide therapy in preventing progression to severe illness in patients at high risk of severe illness and reducing TSR in patients with mild illness.

**Research in context**

**Evidence before this study**

At the time of undertaking this study, SARS-CoV-2 had only been known for a few months. There was one published report that nitazoxanide inhibited the replication of SARS-CoV-2 in cell culture studies. A review of the PubMed online database for “nitazoxanide” and “coronavirus” identified several publications reporting *in vitro* activity of nitazoxanide in inhibiting replication of other coronaviruses and one study reporting the inhibition of IL-6 in mice. A further search for “nitazoxanide clinical trial” identified a large number of clinical trials of nitazoxanide, which identified no significant safety issues associated with its use in humans.

**Added value of this study**

This randomized, double-blind placebo-controlled clinical trial provides evidence that treatment of outpatients with mild or moderate COVID-19 with nitazoxanide 300 mg extended-release tablets administered orally 600 mg twice daily for five days may reduce the risk of progression to severe illness in participants at high risk and the time to sustained recovery in patients with mild illness.

**Implications of all the available evidence**

Further clinical trials with larger numbers are warranted. The availability of a safe, oral, scalable, host-directed therapeutic for mild or moderate COVID-19 could play an important role in reducing the number of severe illnesses and hospitalizations during the ongoing public health crisis.

Alt-text: Unlabelled box

## Introduction

The progress in developing effective vaccines against SARS-CoV-2 infection has been remarkable. Coupling effective therapies with vaccination programs will be critical to controlling the pandemic.^1^ However, the outcome of significant efforts to develop novel therapies, particularly in outpatients, has so far been limited. In the absence of approved alternatives, therapy with nutraceuticals, combinations of intracellular anti-infectives, inhaled/oral corticosteroids, antiplatelet agents/anticoagulants, and supportive care including supplemental oxygen, monitoring and telemedicine have been proposed.^2^ Promising results have been achieved with monoclonal antibodies, yet these treatments are costly, prone to viral resistance, and can only be administered in a clinic setting.^3^, ^4^, ^5^ There is a need for safe, oral medications that can be distributed broadly and in settings with scarce healthcare resources to prevent the worsening of mild or moderate COVID-19 symptoms and subsequent hospitalization.

Nitazoxanide is approved for use in the United States for the treatment of diarrhea caused by *Cryptosporidium parvum* and *Giardia intestinalis* infections and has been used throughout Latin America and Asia for the treatment of intestinal parasitic infections. In the 25 years since nitazoxanide was first introduced, approximately 500 million people have been treated worldwide, and the drug has demonstrated a favorable safety record in both adults and children. Nitazoxanide has been previously shown to be active *in vitro* against a broad range of viruses including MERS and certain animal coronaviruses^6^, ^7^, ^8^ and suppress secretion of cytokines associated with the inflammatory response to respiratory infection.^9^^,^^10^ Recently, nitazoxanide was identified as a candidate drug for SARS-CoV-2 infection based on high throughput screening and *in vitro* virus culture.^11^, ^12^, ^13^, ^14^, ^15^

The antiviral activity of nitazoxanide is attributed to a host-directed mechanism, ultimately targeting the formation of key viral proteins at a post-translational level and stimulating innate immunity.^9^^,^^16^^,^^17^ Studies suggest nitazoxanide has a low potential to select for viral resistance.^17^^,^^18^ Furthermore, multiple studies indicate nitazoxanide has a synergistic effect when combined with other drugs active against SARS-CoV-2.^13^^,^^15^^,^^19^

A recent study has demonstrated *in vitro* that nitazoxanide blocks the spike maturation of the B.1.1.7, P1 and Delta mutants with the same efficacy as for the reference Wuhan strain and D614G mutations^12^ (and paper submitted).

In a multicenter, randomized, double-blind, placebo-controlled trial in patients hospitalized with moderate-to-severe COVID-19, treatment with nitazoxanide 600 mg twice daily for seven days was associated with reductions in rates of mortality and mechanical ventilation, duration of supplemental oxygen and time to hospital discharge compared to placebo.^20^

The multicenter, randomized, double-blind, placebo-controlled trial reported here was designed to evaluate the effectiveness of nitazoxanide 300 mg extended-release tablets administered orally 600 mg twice daily for five days for treatment of outpatients with mild or moderate COVID-19. We hypothesized that treatment with nitazoxanide would reduce the duration of symptoms of mild or moderate COVID-19 compared to treatment with placebo (primary analysis) and the proportion of patients progressing to severe illness compared to placebo (key secondary analysis).

## Methods

### Study design and participants

This was a randomized double-blind placebo-controlled trial conducted in 36 outpatient medical clinics in the U.S. and Puerto Rico in accordance with current good clinical practices and applicable regulations. IntegReview Institutional Review Board (Austin, Texas) reviewed and monitored the study to ensure protection of the rights and welfare of participants. Written informed consent was obtained from each participant or his/her legal guardian prior to participation in the trial, and written assent was obtained from participants who were minors. The clinical trial protocol is included in supplementary materials (ClinicalTrials.gov Identifier: NCT04486313).

Participants at least 12 years of age presenting within 72 h of onset of symptoms of mild or moderate COVID-19 were eligible to participate in the trial. Minimum symptom requirements were: at least two respiratory symptom domains (head, throat, nose, chest, cough) with a score of ≥2 as determined by scoring the InFLUenza Patient-Reported Outcomes (FLU-PRO©)^21^ questionnaire administered at screening (only one domain score required to be ≥2 if pulse rate ≥90 beats per minute or respiratory rate ≥16 breaths per minute), with no improvement in overall symptom severity from the prior day. Key exclusion criteria were: (i) signs or symptoms suggestive of severe COVID-19 including shortness of breath at rest, resting pulse rate ≥125 beats per minute, resting respiratory rate ≥30 breaths per minute, or SpO2 ≤ 93% on room air at sea level; (ii) previous COVID-19 infection, (iii) immunodeficiency; and (iv) pregnant females and sexually active females of childbearing potential not using birth control.

Nitazoxanide was administered as two 300 mg extended-release tablets (600 mg per dose) orally with food twice daily for five days. The dose was selected based upon a dose-range-finding study in patients with influenza.^22^

### Randomization and masking

Eligible participants were centrally randomized using an interactive web response system 1:1 to receive treatment with nitazoxanide or matching placebo tablets. In addition to study medication, all participants received a vitamin B complex supplement (Super B-Complex™, Igennus Healthcare Nutrition, Cambridge, UK) twice daily to mask any potential chromaturia attributed to nitazoxanide. The randomization list was masked to study participants, the sponsor, investigators, study monitors, and laboratory personnel until the database was locked.

Randomization was stratified according to the severity of COVID-19 illness at baseline (mild or moderate), time from onset of symptoms (<36 h or ≥36 h), and whether participants had risk factors for severe illness based on U.S Centers for Disease Control (CDC) criteria current at the initiation of the study (see Supplementary Material for CDC criteria). Moderate illness was defined by resting pulse ≥90 beats per minute and/or resting respiratory rate ≥20 breaths per minute.

### Study procedures

After randomization, eligible participants underwent a physical examination, collection of nasopharyngeal swabs, and blood and urine samples for laboratory safety testing and assessment of SARS-CoV-2 antibody titers. Study drug was dispensed, and participants were followed for 28 days. Participants were instructed to complete electronic diaries recording oral temperature twice daily and symptom severity once daily in the evening for 21 days and were contacted daily by telephone by site staff on study days 2–7 and 28 to verify compliance and screen for progression to severe illness or other complications. Repeat nasopharyngeal swabs were collected on study days 4 and 10. Follow up blood and urine samples for laboratory safety testing and assessment of SARS-CoV-2 antibody titers were collected on study day 22. Participants were allowed to use acetaminophen and/or dextromethorphan for symptom relief along with standard-of-care rescue medications. Adverse events were collected continuously throughout the study and monitored until the events resolved.

In the absence of any patient-reported outcomes instrument validated specifically for collecting data to measure symptoms of COVID-19, symptom data was collected using the FLU-PRO Plus© symptoms questionnaire. The InFLUenza Patient-Reported Outcome Questionnaire (FLU-PRO©) was developed in accordance with psychometric best practices and FDA guidance for the measurement of symptoms of influenza.^21^ Subsequent literature searches and clinical data analyses support the content and construct validity of the instrument for illness caused by non-influenza respiratory viruses including adenovirus, endemic coronaviruses, enteroviruses including rhinoviruses, parainfluenza and respiratory syncytial virus. A literature search conducted prior to initiation of the study supported content validity of the FLU-PRO Plus© with inclusion of loss of taste and loss of smell for measurement of illness caused by SARS-CoV-2 infection. The questionnaire was completed using an electronic diary app downloaded to each participant's smart phone or a provisioned electronic device so that diary entries were time stamped to ensure timely recording, thereby mitigating risks of recall bias.

Nasopharyngeal swab samples collected at baseline, day 4 and day 10 were tested using the Aptima® SARS-CoV-2 assay (Hologic, Inc, San Diego, CA) and ePlex® Respiratory Pathogen Panel (“ePlex RPP”, GenMark, Carlsbad, California). Baseline, day 4 and day 10 nasopharyngeal swab samples positive for SARS-CoV-2 by the Aptima® SARS-CoV-2 assay were subjected to RT-PCR for analysis of quantitative changes in viral load. Blood samples collected at baseline on day 22 were tested for quantitative anti-SARS-CoV-2 antibodies.

### Primary and secondary outcomes

The primary endpoint was time from the first dose to sustained clinical recovery (TSR), a measure of meaningful within-participant symptom improvement developed and validated in participants with influenza infection. The performance characteristics of the FLU-PRO instrument and appropriateness of background levels in participants with SARS-CoV-2 infection were confirmed by blinded analysis of diary data for this study after database lock and prior to unblinding.

The key secondary endpoint was the proportion of participants experiencing progression to severe COVID-19 illness (shortness of breath at rest and SpO_2_ ≤ 93% on room air or PaO_2_/FiO_2_ <300) over 28 days. This definition was selected over a definition including hospitalization due to variability in physician decisions regarding hospital admission.

### Statistical analysis

The statistical analysis plan is included in supplementary materials. Efficacy analyses were based on a modified intention to treat (mITT) population of participants testing positive for SARS-CoV-2 at baseline. All participants receiving at least one dose of study medication were included in the safety analyses.

In the primary analysis, TSR for the nitazoxanide treatment group was compared to that of the placebo treatment group using a stratified Gehan-Wilcoxon test (α=0.05) where stratification followed that used for randomization. Participants without a sustained response recorded were treated as censored as of the last diary completed, except for participants who were hospitalized or died during the study, who were censored at Day 21.

In the absence of prior experience in participants with COVID-19, the sample size was determined based upon data from two prior clinical trials of nitazoxanide in participants with viral respiratory illnesses caused by influenza or rhinoviruses. A sample size of 312 participants (156 per group) was calculated to provide 90% power to detect a statistically significant difference in the survival distributions between the nitazoxanide and placebo groups (Gehan rank test, two-sided α =0.05).

In the key secondary analysis, proportions of participants progressing to severe COVID-19 illness were compared between the treatment groups using a Cochran-Mantel-Haenszel (CMH) test stratified by the randomization strata, and overall odds ratio with 95% confidence interval and p-value were reported.

### Role of funding sources

This research was sponsored by Romark, Tampa, Florida USA. Employees of Romark were responsible for study design, data collection, data analysis, data interpretation and writing the report.

## Results

**Figure 1:**
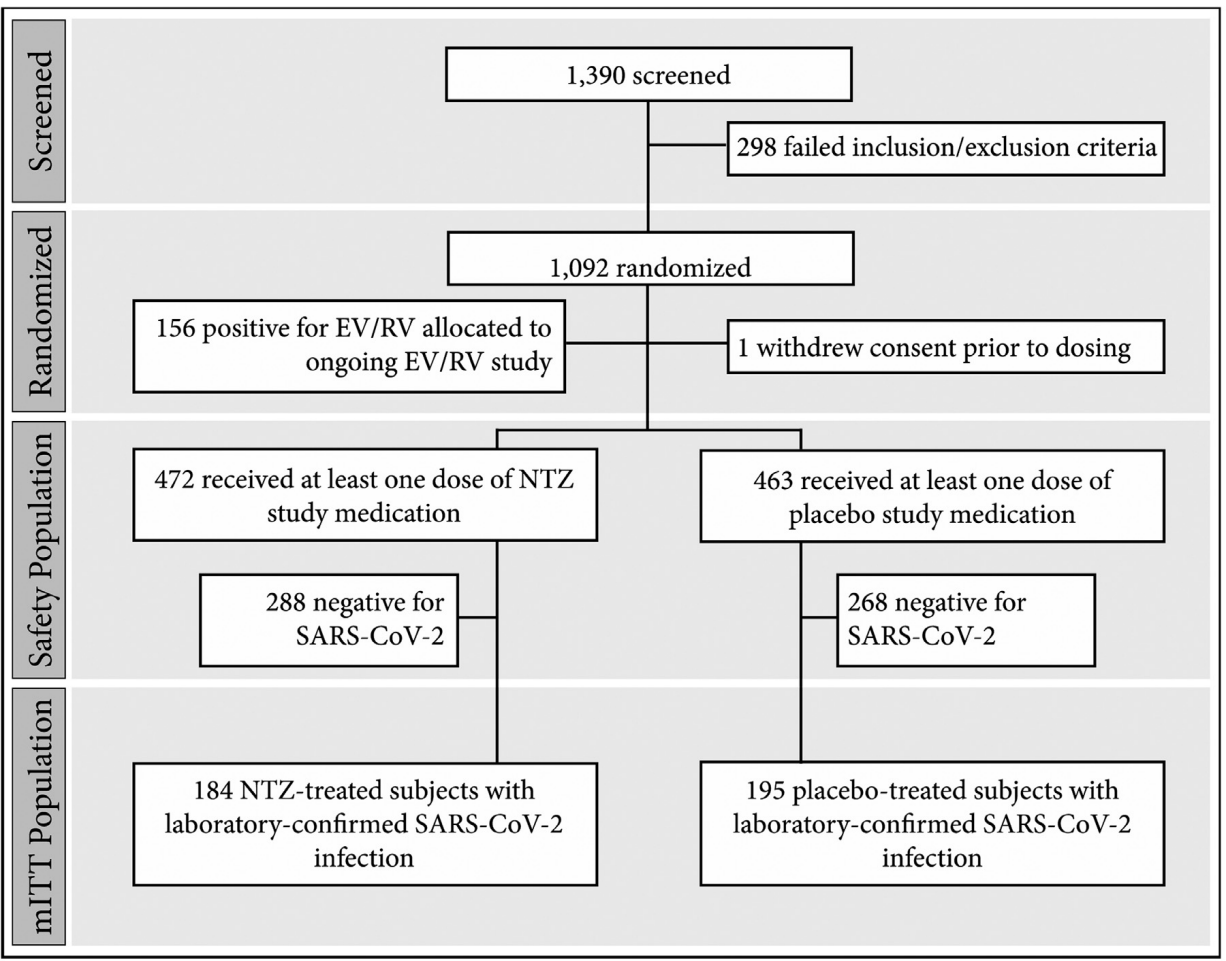
Participant disposition.

From August 18, 2020, to January 8, 2021, 1092 participants were enrolled at 36 sites in the U.S. and Puerto Rico, including 379 with laboratory-confirmed SARS-CoV-2 infection (Figure 1). Demographic and disease-related characteristics of the SARS-CoV-2-infected population included in the efficacy analyses are summarized in Table 1.

**Table 1:** Summary of baseline demographic and disease-related characteristics, mITT population.

	Nitazoxanide (*N* = 184)	Placebo (*N* = 195)	All mITT Participants (*N* = 379)
Male, N (%)	83 (45·1%)	82 (42·1%)	165 (43·5%)
Median (Range) Age (years)	38 (12–83)	42 (13–81)	40 (12–83)
Race or Ethnic Group, N (%)			
White	117 (63·6%)	116 (59·5%)	233 (61·5%)
Hispanic or Latino	59 (32·1%)	71 (36·4%)	130 (34·3%)
Black or African American	4 (2·2%)	4 (2·1%)	8 (2·1%)
Asian	2 (1·1%)	4 (2·1%)	6 (1·6%)
Other	2 (1·1%)	0 (0·0%)	2 (0·5%)
Median (IQR) BMI	28·7 (25·3, 33·5)	29·1 (25·7, 33·6)	28·9 (25·5, 33·5)
Median Hours Since Onset of Symptoms	43·9	46·5	45·9
Moderate Disease Severity, N (%)	68 (37·0%)	65 (33·3%)	133 (35·2%)
At Risk of Severe Illness per CDC Guidelines, N (%)	112 (60·9%)	126 (64·6%)	238 (62·8%)
Viral Load, log_10_ RNA copies/mL			
Median (IQR)	6·38 (4·71, 7·40^1^)	6·34 (4·40, 7·40^1^)	6·36 (4·62, 7·40^1^)
SARS-CoV-2 Antibody-Positive, N (%)	18 (4·7%)	20 (5·3%)	38 (10·0%)

1 7.40 log_10_ copies/mL is the upper limit of quantitation of the assay.

*Primary Outcome*. Ninety-two percent (92%) of all possible daily FLU-PRO questionnaires were completed, and only 22 (5·8%) participants were censored in the primary analysis due to missing diary data prior to the day 22 visit. Median (IQR) TSR were 13·3 (6·3, >21) and 12·4 (7·2, >21) days for the nitazoxanide and placebo groups, respectively (*p* = 0·88).

*Key Secondary Outcome*. Eight participants met the criteria for progression to severe COVID-19, Table 2. 1 of 184 participants (0·5%) treated with nitazoxanide and 7 of 195 (3·6%) treated with placebo experienced progression to severe illness during the 28-day study period, OR [95% CI] 5·6 [0·7–46·1], relative risk reduction 85%, *p* = 0·07, Table 3.

**Table 2:** Participants progressing to severe COVID-19 illness.

Treatment Group	Age Group	Sex	BMI	Viral Load^1^ Day 1/Day 4	Comorbidities	Study Day	Medical Attention Required	Secondary Diagnosis	SpO_2_
NTZ	70–80	M	25·5	7·39 / 4·79	Hypertension, coronary artery disease, atrial fibrillation, bilateral iliac stents, type 2 diabetes mellitus, peripheral vascular disease, hyperlipidemia, decreased renal function, cholecystectomy, prostate cancer with prostatectomy, hypogonadism, gout	5	Hospitalized	COVID-19 pneumonia	91%
Placebo	70–80	M	31·7	6·18 / ≥7·40	Type 2 diabetes mellitus, Parkinson's disease, neuropathy, colon cancer/colon re-sectioning, benign prostatic hyperplasia, obesity	7	Hospitalized	COVID-19 pneumonia	88%
Placebo	60–70	F	36·4	6·54 / 7·06	Hyperlipidemia, osteoarthritis, insomnia, anxiety, obesity	10	Emergency room	COVID-19 pneumonia	93%
Placebo	60–70	M	33·0	ND / ND	Hypertension, hypercholesterolemia, pre-diabetes, gastroesophageal reflux disease, osteoarthritis, anxiety, prostate cancer and surgery, bilateral hip arthroplasty, cataracts, obesity	7	Hospitalized	Dyspnea	88%
Placebo	50–60	M	34·9	6·47 / ND	Hypertension, elevated liver enzymes, previous stroke, subcarinal adenopathy, migraines, nerve issues due to injury, obesity	7	Hospitalized	COVID-19 pneumonia	90%
Placebo	50–60	M	26·8	≥7·40 / NC	Type 2 diabetes mellitus, borderline reduced kidney function, coronary artery disease, coronary artery bypass graft, neuropathy, hypothyroidism, hernias	9	Hospitalized	COVID-19 pneumonia	84%
Placebo	50–60	F	22·8	5·96 / ≥7·40	Hypertension, anxiety, migraine headaches, cigarette use	3	Hospitalized	Syncope	93%
Placebo	30–40	M	35·4	≥7·40 / IND	Essential hypertension, elevated liver enzymes, asthma, obesity	5	Clinic visit	Asthma (exacerbation)	93%

1 log_10_ RNA copies/mL, upper limit of quantitation of the assay was 7·40 log_10_ RNA copies/mL. ^2^ND = Not Done due to insufficient sample; NC = Sample Not Collected (visit missed), IND = Indeterminate result by RT-PCR.

**Table 3:** Analyses of the participants progressing to severe COVID-19 with subgroups based upon different at-risk definitions.

	Placebo	NTZ	OR (95% CI)	P-value	Relative Reduction
All SARS-CoV-2-Positive Participants	7/195 (3·6%)	1/184 (0·5%)	5·6 (0·7–46·1)	0·07	85%
Subgroups:					
“May Be” or “At Increased Risk” for Severe COVID-19 Illness per CDC (protocol-defined stratification factor)	7/126 (5·6%)	1/112 (0·9%)	6·5 (0·8–53·4)	0·05	84%
At Increased Risk for Severe COVID-19 Illness per CDC	7/121 (5·8%)	1/104 (1·0%)	6·3 (0·8–52·3)	0·05	83%
At High Risk of Progressing to Severe COVID-19 and/or Hospitalization (per EUA documents for monoclonal antibodies)^1^	6/69 (8·7%)	1/60 (1·7%)	5·6 (0·7–48·1)	0·08	81%

1 ≥ 65 years of age, BMI ≥35 kg/m2, chronic kidney disease, diabetes, immunosuppressive disease, current receipt of immunosuppressive treatment, or ≥55 years of age with at least one of cardiovascular disease, hypertension, or chronic obstructive pulmonary disease or another chronic respiratory disease.

*Exploratory Outcomes*. 1 of 184 participants (0·5%) treated with nitazoxanide and 6 of 195 (3·1%) treated with placebo experienced hospitalization, emergency room visit, or death during the 28-day follow-up period, OR [95% CI] 4·7 [0·6–39·7], relative risk reduction 82%, *p* = 0·11), Table 4.

**Table 4:** Analyses of the participants experiencing hospitalization, emergency room visit or death with subgroups based upon different at-risk definitions.

	Placebo	NTZ	OR (95% CI)	P-value	Relative Reduction
All SARS-CoV-2-Positive Participants	6/195 (3·1%)	1/184 (0·5%)	4·7 (0·6–39·7)	0·11	82%
Subgroups:					
“May Be” or “At Increased Risk” for Severe COVID-19 Illness per CDC (protocol-defined stratification factor)	6/126 (4·8%)	1/112 (0·9%)	5·6 (0·7–46·8)	0·08	81%
At Increased Risk for Severe COVID-19 Illness per CDC	6/121 (5·0%)	1/104 (1·0%)	5·4 (0·6–45·4)	0·09	81%
At High Risk of Progressing to Severe COVID-19 and/or Hospitalization (per EUA documents for monoclonal antibodies)^1^	5/69 (7·2%)	1/60 (1·7%)	4·6 (0·5–40·6)	0·13	77%

1 ≥ 65 years of age, BMI ≥35 kg/m2, chronic kidney disease, diabetes, immunosuppressive disease, current receipt of immunosuppressive treatment, or ≥55 years of age with at least one of cardiovascular disease, hypertension, or chronic obstructive pulmonary disease or another chronic respiratory disease.

94 and 70% of participants tested positive for SARS-CoV-2 RNA in nasopharyngeal swabs collected at study days 4 and 10, respectively. Qualitative and quantitative tests to detect SARS-CoV-2 were not significantly different between the treatment arms at these time points, Table 5.

**Table 5:** Virologic data by time point.

Time Point	NTZ			Placebo		
	N Samples Analyzed	Percent Positive	Mean (SD) Viral Load Change from Baseline (log_10_ copies/mL)	N Samples Analyzed	Percent Positive	Mean (SD) Viral Load Change from Baseline (log_10_ copies/mL)
Day 4	177	94%	−0·70 (1.573)	178	94%	−1·02 (1·668)
Day 10	177	73%	−2·49 (1.582)	182	71%	−2·61 (1·604)

**Figure 2:**
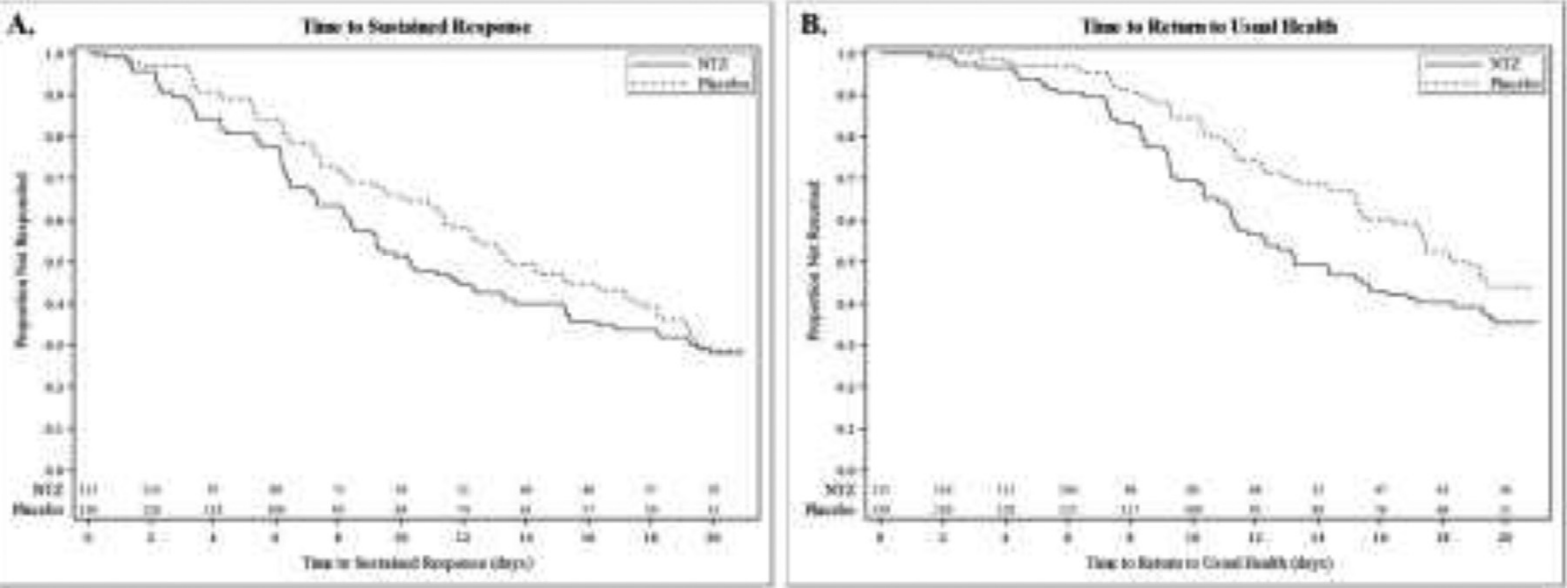
Clinical recovery in participants with mild illness.

In the pre-defined stratum of 246 participants (65%) with mild illness at baseline, participants treated with nitazoxanide experienced a 3·1-day reduction of median TSR (median [IQR] = 10·3 days [6·2, >21] for nitazoxanide [*n* = 116] compared to 13·4 days [7·4, >21] for the placebo group [*n* = 130], *p* = 0·09) and a 5·2-day reduction of median time from first dose until the participants reported returning to usual health (median [IQR] = 13·2 days [9·2, >21] for nitazoxanide compared to 18·4 days [11·4, >21] for the placebo group, *p* < 0·01), Figure 2. In the pre-defined stratum of 133 participants (35%) with moderate illness at baseline, participants treated with nitazoxanide experienced a longer TSR and time to return to usual health. None of the 68 participants with moderate illness in the nitazoxanide arm experienced progression to severe illness while two of 65 in the placebo arm did.

*Safety.* Nitazoxanide was safe and well tolerated. Sixty-three participants (13·3%) treated with nitazoxanide and 75 (16·2%) treated with placebo reported at least one adverse event, predominately classified as mild or moderate in severity and unrelated or possibly related to study drug. Only diarrhea was reported in ≥2% in any treatment group (*n* = 16 [3·4%] in the nitazoxanide group and *n* = 10 [2·2%] in the placebo group). Frequency, severity, and assessment of relationship to study drug of adverse events were similar across treatment groups. Two participants treated with nitazoxanide and seven receiving placebo reported serious adverse events, all unrelated to the study drug. Two participants (both in the nitazoxanide treatment group) died during the study, one due to severe COVID-19 and the other (SARS-CoV-2 negative) secondary to aspiration 19 days after completing therapy. Neither event was considered related to study medication in the judgment of the Investigator, Sponsor, Medical Monitor, and Independent Data Monitoring Committee. Two nitazoxanide-treated participants and three receiving placebo discontinued study medication due to adverse events.

## Discussion

The COVID-19 pandemic continues with surges caused by SARS-CoV-2 variants, and hospitalizations posing a major threat to health care systems in many countries. Despite the development of vaccines which are now being distributed, widespread vaccination will need time to be implemented worldwide and will not fully prevent infection. Thus, there is a critical need for a safe, easy-to-administer antiviral therapeutic that can be distributed through pharmacies and administered early for treatment of mild or moderate COVID-19.

We report a multicenter randomized double-blind placebo-controlled trial conducted at 36 outpatient centers in the United States and Puerto Rico between August 2020 and February 2021. The study employed a concurrent placebo control and enrolled a broad range of participants at least 12 years of age, 63% of whom had risk factors placing them at higher risk of severe COVID-19. Participants were enrolled based upon symptoms to ensure early treatment, avoiding limitations associated with availability and delays in diagnostic testing, and 379 participants with confirmed SARS-CoV-2 infection were analyzed for effectiveness. The trial was appropriately blinded, and participants were closely followed for 28 days. The trial endpoints were objective and well-defined, and rigorous data collection procedures were employed.

This trial was designed early in the pandemic when information to guide selection of endpoints and patient populations and access to reliable and timely diagnostic testing were limited. The endpoints and patient population were selected based upon the design of historical clinical trials in outpatients with uncomplicated influenza. To ensure early treatment, participants were enrolled based upon symptoms, and nasopharyngeal swabs were collected at the baseline visit to identify participants with laboratory-confirmed SARS-CoV-2 infection to be included in the analyses of the modified intent-to-treat population.

In late 2020, weeks before the present study was fully enrolled, exploratory analyses from early studies of the monoclonal antibodies, bamlanivimab and casirivimab plus imdevimab, in treating mild or moderate COVID-19 led to adoption of an efficacy endpoint focused on proportions of emergency room visits or hospitalizations within 28 days. The observed proportions experiencing hospitalization or death in those studies were 1/101 (1.0%) for bamlanivimab (700 mg)-treated participants compared to 9/156 (5.8%) for placebo-treated participants (*p* = 0·09), an 83% relative reduction, and 4/215 (1·9%) for casirivimab/imdevimab (2400 mg)-treated participants compared to 10/231 (4·3%) for placebo-treated participants (*p* = 0·18), a 57% relative reduction. In November 2020, these products were made available under emergency use authorizations in the United States based on this data.^3^^,^^4^^,^^23^^,^^24^ Larger follow-on studies of these and other monoclonal antibodies have subsequently confirmed their effectiveness with observed reductions in COVID-19-related hospitalizations or deaths compared to placebo in the range of 70%. In those follow-on studies, the endpoint used to evaluate therapeutics for treating mild or moderate COVID-19 has evolved to proportions of participants experiencing hospitalization or all-cause death within 28 days, and patient populations have focused on participants with risk factors for severe illness.

In the present clinical trial, the pre-defined primary analysis of TSR in all participants with mild or moderate COVID-19 illness did not detect a significant difference between the nitazoxanide and placebo treatment groups. In the pre-defined stratum of participants with mild illness at baseline, however, treatment with nitazoxanide was associated with a 3·1-day reduction of median TSR (median [IQR] = 10·3 days [6·2, >21] for nitazoxanide [*n* = 116] compared to 13·4 days [7·4, >21] for the placebo group [*n* = 130], *p* = 0·09), and this finding was supported by a 5·2-day reduction of median time from first dose until the participants reported returning to usual health (median [IQR] = 13·2 days [9·2, >21] for nitazoxanide compared to 18·4 days [11·4, >21] for the placebo group, *p* < 0·01). Given present knowledge of the stages/phases of COVID-19 disease and their importance in making treatment decisions, this data suggests that the TSR endpoint may be more relevant for patients with mild illness where there may be less between-patient variability of the illness and where damage to the respiratory tract is minimal at the onset of treatment.

In the key secondary analysis, 1 of 184 participants (0·5%) treated with nitazoxanide 600 mg orally twice daily for five days experienced progression to severe illness during the 28-day study period compared to 7 of 195 (3·6%) treated with placebo (OR [95% CI] 5·6 [0·7–46·1], relative risk reduction 85%, *p* = 0·07). All severe illnesses were clinically meaningful with six of the eight requiring hospitalization. Each of the eight severe illnesses occurred between study days 3 and 10 in participants at high risk of severe illness according to CDC criteria. This trial was not designed with adequate statistical power for this analysis. Nevertheless, while the numbers of observed events are low, they are quantitatively and qualitatively similar to those used to support emergency use of the first monoclonal antibodies for treating mild or moderate COVID-19.^3^^,^^4^^,^^23^^,^^24^

We did not observe differences between treatment groups in qualitative or quantitative SARS-CoV-2 RNA in nasopharyngeal swabs on study day 4 or 10. While others have reported modest reductions of quantitative or qualitative RNA in nasopharyngeal swabs at different points after the end of treatment with nitazoxanide,^20^^,^^25^^,^^26^ the methods used for collection, sample handling and measuring viral loads from nasopharyngeal swabs in large multi-center clinical trials have not been validated or shown to be predictive at the patient- or trial-level of viral load, inflammation or symptoms in the lungs or clinical outcomes. It is also unclear whether RT-PCR accurately measures infectious virus, since viral RNA may persist for some time, even in the absence of replication-competent virus. This may be a particularly important limitation in the context of a host-directed therapeutic like nitazoxanide that affects assembly of the virus.

In this study, nitazoxanide was safe and well tolerated, consistent with its well-established safety profile. Safety will be an important attribute for a therapeutic for mild or moderate COVID-19.

This randomized, double-blind, placebo-controlled clinical trial provides evidence that early treatment with nitazoxanide may reduce the progression to severe illness in high-risk participants and the time to sustained clinical response in participants with mild illness. Larger trials with adequate statistical power are being undertaken to confirm these hypotheses. In view of observed synergies *in vitro*,^13^^,^^15^^,^^19^ nitazoxanide may also be considered as a component of combination therapy with direct-acting antiviral agents.

The availability of a safe, oral, scalable, host-directed antiviral for early treatment of COVID-19 could play an important role in reducing the number of severe illnesses and hospitalizations during this ongoing major public health crisis.

## Declaration of interests

JFR is an employee of and owns equity interest in Romark, L.C. MB, JF and DM are employees of Romark, L.C.; CB is an adviser for Romark, L.C.

## Appendix Supplementary materials

Image, application 1

Image, application 2

Image, application 3
