# Supplementary material for: A randomized double-blind placebo-controlled clinical trial of nitazoxanide for treatment of mild or moderate COVID-19
Source: eClinicalMedicine. 2022 Feb 28;45:101310. doi: 10.1016/j.eclinm.2022.101310 (PMC8883002; doi:10.1016/j.eclinm.2022.101310)
Supplement: Supplementary file 1 [file mmc1.docx]

**Supplementary Material**

**Vanguard Study Team**

1. Maher Agha, MD (OnSite Clinical Solutions – Charlotte, Charlotte, NC)
2. Ayoade Akere, MD (Eagle Clinical Research, Chicago, IL)
3. Ali Bajwa, MD (Centex Studies – Westfield, Houston, TX)
4. Greg Bostick, MD (Cullman Clinical Trials, Cullman, AL)
5. Jose F. Cardona, MD (Indago Research & Health Center, Inc., Hialeah, FL)
6. Ivan Carreras, MD (Clintex Research Group, Inc, Coral Gables, FL)
7. Jorge Diaz, DO (Doral Medical Research, Inc., Hialeah, FL)
8. Dina Doolin, DO (Riverside Clinical Research, Edgewater, FL)
9. Timothy Elder, MD (SIMEDHealth, LLC, Gainesville, FL)
10. Almena L. Free, MD (Pinnacle Research Group, LLC, Anniston, AL)
11. Bernard Garcia, MD (Invesclinic U.S., Ft. Lauderdale, FL)
12. Hiram Garcia, MD (Rio Grande Valley Clinical Research Institute, Pharr, TX)
13. Darin M. Gregory, MD (Pioneer Clinical Research, Bellevue, NE)
14. Barry Heller, MD (Long Beach Clinical Trials, Long Beach, CA)
15. Rubaba Hussain, MD (Prime Global Research, Bronx, NY)
16. Talal Khader, MD (Vida Clinical Studies, Dearborn, MI)
17. Rogelio Machuca, MD (Machuca Family Medicine, Las Vegas, NV)
18. Eric J. Melvin, MD (Clinical Trials of America, LLC, Mt. Airy, NC)
19. Randall P. Miller, MD (Horizon Research Group of Opelousas, LLC, Eunice, LA)
20. Nidal Morrar, MD (G & L Research, Foley, AL)
21. Joshua B. Oaks, MD (Progressive Clinical Research, Bountiful, UT)
22. Arin Piramzadian, DO (OnSite Clinical Solutions, Charlotte, NC)
23. Joe E. Pouzar Jr., MD (Centex Studies- Houston, Houston, TX)
24. Michael J. Rankin, MD (Worthington Urgent Care, Worthington, OH)
25. Ramon Reyes, MD (BFHC Research, San Antonio, TX)
26. Patricia D. Salvato, MD (Diversified Medical Practices, Houston, TX)
27. Jodi Sanson, MD (HealthStar Research, Hot Springs, AR)
28. Pantea Shoja, MD (Pearl City Urgent Care, Pearl City, HI)
29. Javier Sosa, MD (Hospital San Cristobal, Ponce, PR)
30. Alan Tannenbaum, MD (Vanguard Clinical Research, LLC, Fort Myers, FL)
31. Rafaelito Victoria, MD (Atella Clinical Research, La Palma, CA)
32. Kishor Vora, MD (Research Integrity, LLC, Owensboro, KY)
33. George S. Walker, MD (Best Clinical Trials, New Orleans, LA)
34. David Wever, MD (Cahaba Research- Pelham, Pelham, AL)
35. Michael Yuryev, DO (Integrative Clinical Trials, LLC, Brooklyn, NY)
36. Jeffrey Zacher, MD (West Valley Research Clinic, Phoenix, AZ)

**Definition of Subjects at Risk of Severe Illness (per CDC):**

Subjects who are ≥65 years of age; subjects with COPD, Type 2 diabetes mellitus, obesity (BMI ≥30), chronic kidney disease, sickle cell disease, serious heart conditions (such as heart failure, coronary artery disease, or cardiomyopathies), asthma (moderate or severe), cerebrovascular disease, cystic fibrosis, hypertension or high blood pressure, immunocompromised state (due to immune deficiencies, HIV, use of corticosteroids, or use of other immune-weakening medications), neurologic conditions (e.g., dementia), liver disease, pulmonary fibrosis, past or present history of smoking, thalassemia, or type 1 diabetes mellitus.
