# Supplementary material for: A randomized double-blind placebo-controlled clinical trial of nitazoxanide for treatment of mild or moderate COVID-19
Source: eClinicalMedicine. 2022 Feb 28;45:101310. doi: 10.1016/j.eclinm.2022.101310 (PMC8883002; doi:10.1016/j.eclinm.2022.101310)
Supplement: Supplementary file 2 [file mmc2.docx]

**Captions for Supplementary Material**

| **File Name** | **Caption** |
| --- | --- |
| Supplementary Material.docx | Supplementary Material |
| RM08-3008 Protocol v4.0 FINAL_Redacted.pdf | Protocol |
| Romark RM08-3008 Statistical Analysis Plan Version 1.2_Redacted.pdf | Statistical Analysis Plan |
